# Effect of Large Yellow Croaker By-Products on Physical Properties and Thermal Gelling Properties Changes in Reconstituted Surimi Gel

**DOI:** 10.3390/foods14111949

**Published:** 2025-05-30

**Authors:** Fen Zhou, Fengchao Wu, Xiaoqing Ren, Jiaxin Guo, Xichang Wang

**Affiliations:** 1 College of Food Science and Biotechnology, Tianjin Agricultural University, Tianjin 300392, China; wufengchao2023@163.com (F.W.); xiaoqingren@tjau.edu.cn (X.R.); guojiwxin@163.com (J.G.); 2 College of Food Science and Technology, Shanghai Ocean University, Shanghai 201306, China; 3 National Bulk Freshwater Fish Processing Technology R&D Sub-Center, Tianjin 300392, China

**Keywords:** large yellow croaker, taste substances, surimi gels, physical properties, thermal gelling properties

## Abstract

To investigate the effects of water-soluble taste substances (WSTSs) on the physical properties and thermal coagulation properties of reconstituted surimi gels, this study used large yellow croaker muscle (FM) and the WSTS from by-product minced meat (MM) (skin, tail, and head meat (HM)). It was observed that these exogenous additions could effectively improve the surimi gel’s whiteness, gel strength and umami amino acid content. When these were added, the relaxation times of bound water in FM, MM and HM groups were shorter in the 10% exogenous addition treatment, and the surimi particle size (D10, D50, D90, d4, 3, d2, 3) was smaller. This implies a correlation between the WSTS and the moisture preservation capacity of recombinant surimi gels, whereby WSTS facilitates the cross-linking of protein molecules, leading to the formation of a densely interconnected network architecture. This research can provide theoretical guidance for the processing of surimi gel combined fish flavor substances and freshwater surimi, thereby improving the flavor characteristics of freshwater surimi gel.

## 1. Introduction

Surimi, renowned for its unique texture, delicious flavor, and high nutritional value due to low cholesterol and fat content, can be transformed into diverse product formats, including fish balls, paupiette, and fish cakes [1]. In order to optimize the use of aquatic resources, the production of surimi frequently leverages lower-grade raw materials or combines inferior surimi with its superior counterpart [2]. There is an essential need to broaden the existing market scope and foster new avenues for the production of superior surimi-derived culinary items that elevate consumer satisfaction.

Myofibrillar proteins, being the primary constituents of surimi, have a critical function in the heat-induced gelation process of surimi [3]. In fish, muscle tissues contain different amounts of myosin, troponin, endogenous glutamine aminotransferase, glutamic acid, and lysine, which promote the cross-linking of surimi in the gelation process. In addition, related studies have indicated that the gel properties of seawater surimi are better than those of freshwater surimi, so the fish type affects the gel properties of surimi.

As the techniques for the artificial propagation and breeding of large yellow croaker, *Pseudosciaena crocea* (*P. crocea*), have evolved and matured, it has turned into one of the most abundant marine fish species in China. Its flavor and nutritional value have garnered the deep appreciation of both local and global consumers [4,5]. Recombinant products offer the possibility of adding various ingredients, giving a specific taste, texture and function. As raw materials for surimi products, freshwater fish resources are relatively abundant, while marine fish resources have been significantly decreased [6,7]. This is helpful in further utilizing the freshwater fish to produce surimi products with the characteristics of marine fish textures and flavors. The freshwater fish, characterized by high yield, high protein content and numerous small fishbones, are typically processed into surimi to enhance product value and improve the consumer’s eating experience [8]. The by-products such as fish bone, skin, head, and viscera are often discarded in fish processing industries. These by-products contain minerals, proteins, fats, calcium, phosphorus and flavor substances. Significantly, digestive organs serve as potential reservoirs for a variety of enzymes, including lipases and proteases [9]. The gel-forming capacity of reconstituted surimi can be improved by additives, mainly polysaccharides, inorganic salts and organic acids, or by adjusting pH, ionic strength, temperature and component level [10,11].

Physicochemical properties including color, texture and gel properties are important for evaluating surimi quality and consumer acceptance [12]. In this study, large yellow croaker muscle (FM) and the WSTSs from by-product minced meat (MM) comprising skin, tail, and fish head meat (HM) were used as surimi gel production materials. The textural properties, color, taste substances and water distribution were analyzed to investigate the effects of WSTS on the physical properties and thermal coagulation properties of reconstituted surimi gels.

## 2. Materials and Methods

Fresh fish (*P. crocea*) was sourced from Qimin Agriculture and industry Co., Ltd., Ningde, China. The FM was finely ground before application. MM was extracted from the skin and tail, while the meat from the fish head yielded HM. Commercially available frozen silver carp surimi (AAA grade, Hubei Meister Food Co., Ltd., Jingzhou, China, possessing 75.65% moisture, 14.26% protein, and 0.49% fat content) was utilized as the surimi input. This frozen surimi was segmented into roughly 500 g units, vacuum-packaged, sealed, and kept at −20 °C until required.

### 2.1. Water-Soluble Taste Substances Preparation

**Figure 1:**
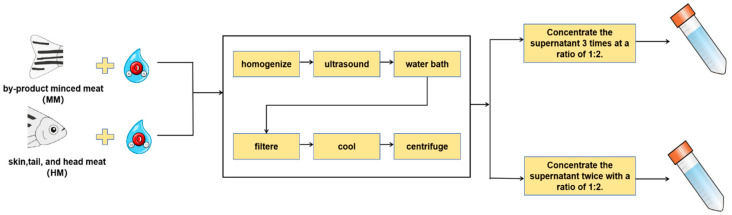
WSTS extraction flowchart.

The procedure for the extraction of WSTS was conducted via the technique outlined by Zhou and Wang [13]. Samples and ultrapure water were combined at a 1:2 (g/mL) ratio and then homogenized for 30 s. The blend was then subjected to 10 min of ultrasound before being immersed in a water bath maintained at 100 °C for a duration of 2 h. Following this, the mixtures were carefully filtered using a Whatman No. 54 filter membrane to ensure purity. The filtered solutions obtained were allowed to cool and subsequently centrifuged (10,621× *g*, 20 min, 4 °C). The clear liquid gathered above the residue, referred to as the supernatant, was then isolated and concentrated. This concentrated solution represents the water-soluble taste substances. The concentration process followed a 1:2 (g/mL) ratio with the original filtrate. The MM and HM groups refer to the substances concentrated three and two times, respectively (as depicted in Figure 1). As shown in our previous study [13], these conditions yield umami amino acid contents similar to that in the original unextracted sample.

### 2.2. Surimi Gel Preparation

**Figure 2:**
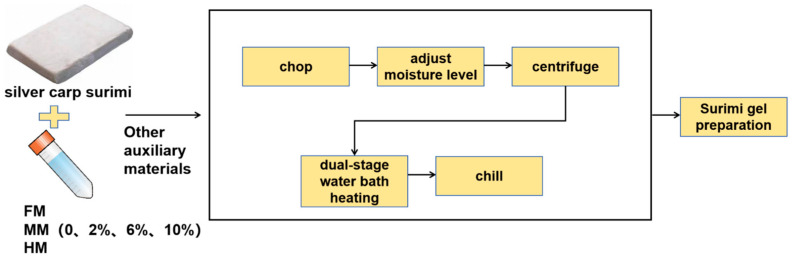
Flowchart of surimi gel preparation process.

The surimi gel was fabricated based on Zhou and Wang’s method [14]. The silver carp surimi was defrosted at 4 °C over 4 h and then diced into cubes. The cubes were then chopped to 1377 g for one min, followed by the addition of 3.00% (*w*/*w*) salt and 5.00% (*w*/*w*) iced deionized water, and subsequent mincing for another minute. A further addition of 5.00% (*w*/*w*) corn starch was followed by an extra minute of chopping. The surimi was then mixed with WSTS and FM in proportions of 2.00%, 6.00% and 10.00% (relative to the surimi’s wet weight), and chopped once more for a minute to achieve a homogenized paste. The moisture level was ultimately adjusted to reach 80%. Approximately 35 g of the samples were dispensed into 50 mL tubes, sealed securely, and then centrifuged at a speed of 956 g for 5 min at 4 °C, ensuring the expulsion of any lingering air bubbles. To enhance the gel properties, a dual-stage water bath heating methodology was employed. This method helps in prolonging the gelation process while also minimizing the chance of gel degradation, as explained in research by Wang et al. [15]. This heating sequence comprised a 40 °C water bath immersion for an hour, succeeded by a 90 °C bath for 20 min. Subsequently, the samples were chilled in an ice-water bath for half an hour (as depicted in Figure 2). The control group comprised surimi gels that were not infused with any WSTS or FM.

### 2.3. Whiteness Evaluation

A colorimeter (Minolta Chroma Meter CR-400, Japan) was employed to determine the whiteness of both the surimi and the surimi gel samples. Lightness (L*), redness/greenness (a*), and yellowness/blueness (b*) values were logged. Prior to usage, the colorimeter was calibrated with a white standard plate. The whiteness (W) was computed as per the following equation [16]:

W = 100 − [(100 − L*)^2^ + a*^2^ + b*^2^]^1/2^ (1)

### 2.4. Particle Size Measuremen

All surimi samples underwent particle size determination using a BT-9300ST laser particle size analyzer (a precision instrument originating from Dandong City, Liaoning Province, China). This high-resolution tool provides reliable and accurate measurements of particle dimensions. The samples were diluted by distilled water in a ratio of approximately 1:50. Then, 1 mL of 1% SDS was added and the mixture was homogenized at 956 g for 30 s to fully disperse the surimi. The particle size was measured in the range 0.1–1000 μm. The refractive index of the particles was 1.520 and the obscuration rate was 16.63%, with water (having an obscuration rate of 1.33) serving as the medium [17].

### 2.5. Textural Properties Analysis

Post-heating, the samples were shaped into cylindrical gels, each 20 mm in diameter, and a selection of six specimens were set aside for evaluating texture profile analysis (TPA) and gel strength. The TPA was conducted using a Stable Micro Systems’ Model TA-XT2i texture analyzer, a highly precise instrument based in Surrey, England. Each gel sample was subjected to dual compression cycles until they were reduced to 30% of their original height, with the process involving a P/50 probe. The compression stages involved a pre-test speed of 2 mm/s, a test speed of 1 mm/s, and a post-test speed of 1 mm/s.

The determination of gel strength was accomplished according to the technique proposed by Yang and Meng, albeit with minor modifications [18]. The Model TA-XT2i texture analyzer was again employed for this purpose. A P/5S cylinder probe was used to apply steady pressure onto the sliced surface of the gel samples at a consistent depression speed. The probe compressed each sample over a 15 mm distance, maintaining a compression speed of 2 mm/s. The breaking force (g) and the corresponding deformation (mm) were subsequently documented. The gel strength was then computed using the formula: Gel strength (g*mm) = breaking force(g)* deformation (mm).

### 2.6. Umami Amino Acid Evaluation

The determination of umami amino acid concentration within gel samples was carried out in alignment with the methodology proposed by Chen et al. [19]. A set of standard amino acid samples was sourced from Sigma (Sigma Chemical Co., St. Louis, MO, USA). Precisely 2.00 g (±0.01 g) of each sample was measured and transferred to a 50 mL centrifuge tube, followed by the addition of 15 mL of 5% Trichloroacetic acid for thorough amalgamation. After a rest period of 2 h, the samples were subjected to a 5 min ultrasonication. The resulting supernatant was then separated via centrifugation at 4 °C, spinning at 10,621× *g* per minute for 10 min. The next step involved pH adjustment, specifically, 5 mL of the separated supernatant was adjusted to a pH of 2.0 using 6 mol/L NaOH and 1 mol/L NaOH. To reach a final volume of 10 mL, ultrapure water was added, and a 1 mL aliquot was passed through a 0.2 μm membrane filter. Quantitative analysis was carried out using an L-8800 amino acid analyzer from Hitachi (Chiyoda, Japan).

### 2.7. Low-Field NMR Spin–Spin Relaxation (T_2_) Measurements

The assessment of NMR relaxation was carried out by deploying a Niumag Benchtop Pulsed NMR Analyser PQ001 (Niumag Electric Corporation, Shanghai, China), an instrument designed to resonate at a frequency of 21 MHz for protons. This was performed on both unheated treatments and the heated gel sample, with approximately 5.00 g of each sample placed on the NMR platform. Utilizing the Carr–Purcell–Meiboom–Gill sequence, the spin–spin relaxation time (T_2_) was accurately determined. Various relaxing time constants (T_2b_, T_21_, T_22_, and T_23_) were derived through a multi-exponential fitting model facilitated by MATLAB^®^ software 2017 (MathWorks, Menedik, Natick, MA, USA). During this process, corresponding water proportions (P_2b_, P_21_, P_22_, and P_23_) were also documented. Information from 4000 echoes was collated from 8 scan iterations, and each measurement was repeated thrice to ensure data accuracy and consistency.

### 2.8. Sensory Evaluation

The sensory evaluation of surimi products was conducted using the quantitative descriptive analysis method. Twenty sensory evaluators from the professional-trained sensory evaluation panel in our laboratory were selected for the sensory assessment. First, sensory evaluations were performed on surimi products with large yellow croaker muscle added at levels of 0.00%, 2.00%, 6.00%, and 10.00%. The surimi product with the highest sensory score corresponding to the addition level was selected as the “standard product” (indicating it has the taste characteristics of large yellow croaker and is favored by consumers). For other treatment groups, scores were sequentially assigned by taking the scores of each evaluation indicator of the “standard product” as the judgment criteria.

### 2.9. Statistical Analysis

Significant differences between means (*p* < 0.05) were evaluated through Duncan’s multiple range test by one-way ANOVA using SPSS 19.0, and the results are presented as the means ± SD. Graphical representations were plotted using Sigma Plot software (Version 12.0; Systat Software Inc., San Jose, CA, USA).

## 3. Results and Discussion

### 3.1. Whiteness of Reconstituted Surimi and Surimi Gel

**Figure 3:**
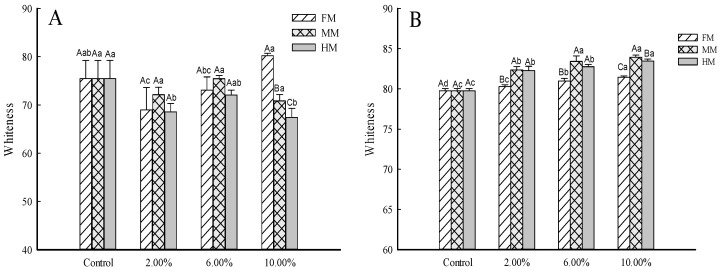
Analysis of reconstituted surimi and surimi gel whiteness. ***Note:*** Mean values of six replicates and standard deviation. (**A**) Surimi sample. (**B**) Surimi gel sample. FM: large yellow croaker muscle. MM: water-soluble substance concentrated three times from fish skin and fish tail minced meat. HM: the water-soluble substance concentrated two times from fish head meat. a–c indicate significant differences among the additions of same additive (*p* < 0.05) according to Duncan’s test. A–C indicate significant differences among the additives at the same addition (*p* < 0.05) according to Duncan’s test. The same is shown below.

Whiteness, which affects consumer acceptance, is considered a crucial factor in surimi quality [20]. The color of surimi before gelation indirectly affects the color of surimi gel. Changes in the whiteness of surimi and surimi gel at different levels of by-product addition are shown in Figure 3A,B. With the increase in FM, MM and HM, the whiteness of surimi gel gradually increased. Importantly, for the same level of addition, the trend was MM > HM > FM. For surimi (Figure 3A) with 10.00% FM, the whiteness was greater than in other groups, while the MM group and HM group showed a trend of first decreasing, then increasing, and finally decreasing again. Both the MM and HM were in an aqueous solution state and affected the whiteness of surimi. During the chopping process, the protein absorbed water and swelled, and the water content on the surface loosened by the protein was relatively high, increasing the light scattering ability of the surimi [21]. The flavoring substances (MM and HM) at multiple concentrations contributed to inherent pigmentation that affected the final color of surimi with increases in the added amount.

Moreover, the gels’ enhanced whiteness compared to the surimi represents a notable observation. This phenomenon might be attributed to the thermal denaturation of proteins during surimi gel preparation, leading to the formation of a more tightly knit network. Such a dense arrangement could increase light scattering, as suggested by Singh and colleagues in their 2020 study [22]. These results may also be related to the different physical states of the additives; the protein–water interaction increased during gelation, enhancing the light scattering on the gel surface.

### 3.2. Dispersion Characteristics of Particles in Reconstituted Surimi Analysis

The particle size and distribution in the surimi directly influence the quality of the intermediate or final product, and are therefore important variables. Table 1 presents the particle sizes corresponding to D10, D50, D90, d4, 3 and d3, 2 in the FM, MM, and HM groups of reconstituted surimi. It can be observed that the particle size in the reconstituted surimi initially increased and then decreased with an increase in the added amount. In the FM group, the addition of 6.00% significantly increased the particle diameters, which reached the maximum (*p* < 0.05). In the MM and HM groups, the maximum was attained when the addition was 2.00%, except for the particle size corresponding to D90 in the HM group. Furthermore, the particle sizes in the FM group were significantly larger than those in the MM and HM groups (*p* < 0.05).

In the FM group, the muscle mince and surimi were thoroughly mixed during the mincing process, and wrapped or embedded to form larger surimi particles, thereby increasing the size of the reconstituted surimi particles. With the increased addition of FM, the fat content of the reconstituted surimi increased (7.92% in muscle, 0.49% in frozen surimi). The fat allowed for the better wrapping or a mosaic pattern of the protein–fat–protein in the reconstituted surimi, making the surimi relatively delicate, and contributing to a decrease in the particle size of the reconstituted surimi [23]. Both MM and HM groups contained water-soluble flavor compounds, and protein hydration occurred during chopping, causing the protein to absorb water and swell, thus increasing the surimi particle size. With further addition, the surimi became more tender and the particle size decreased during the chopping process. Prominent in the field is the understanding that a well-dispersed protein structure aids in protein stretching and revealing reactive groups. This can foster protein intermolecular interactions during the gelation process, in turn bolstering the strength of surimi gels [24].

### 3.3. Textural Properties Analysis of Reconstituted Surimi

TPA serves as a valuable tool in assessing the structural characteristics and inherent properties of surimi gels [25]. The gel texture analysis simulates the chewing process to compress the test sample and obtain parameters corresponding to a sensory evaluation. The hardness reflects the “biting force” or “pinching force” required to collapse the gel network structure once the gel is compressed by a trigger force. Deemed as a vital indicator of cooking quality, springiness reflects the capacity of the gel to revert to its initial state after an external force has caused deformation, all within a specified time frame. Gumminess is the essential characteristic of the semi-solid state of the gel; it is defined as the extent to which the sample could be ruptured before being deformed. Chewiness reflects the chewability quality of surimi gels [26].

Regarding the textural properties of the different gels (hardness, springiness, gumminess and chewiness), results are shown in Table 2. Changes in elasticity were not significant in all groups (*p* > 0.05). The hardness, gumminess and chewiness of the FM group all increased with the increase in FM addition; this result is consistent with that for surimi gel strength. A reduction in hardness might be linked to a corresponding decrease in the structural integrity of the surimi–gel network [20]. The hardness, cohesiveness and chewiness all decreased when the addition reached 2.00% in both the MM group and the HM group when they were added with water-soluble taste substances. Subsequently, they all increased with increasing additions of MM and HM, and were all larger than in the control group. The overall results were FM > MM > HM. These results indicate that the addition of FM, MM and HM could improve the texture characteristics of the gel. Surimi by-product hydrolysates (SBPHs) were added in Zhang’s paper, and the results show that the addition of SBPHs improved the water holding capacity, texture property, and gel characteristic of the surimi gel, and reduced the cooking loss and aperture area [27].

As a rule of thumb, surimi gels that demonstrate enhanced gel strength often appear to be more appealing and appetizing to the consumer base [15]. Table 2 also illustrates thegel strength of the FM, MM and HM, respectively. The gel strength increased for samples with higher levels of FM, MM and HM. At the same amount of addition, the samples could be ranked as FM > HM > MM. The gel strength of proteins containing fillers may depend on the characteristics of the exogenous additives, which could enhance or inhibit the gel network structure. In addition, the gel strength is closely related to the expressible drip. The lower the juice loss, the stronger the water-holding capacity of the gel network structure, the denser the gel network space, and the stronger the gel strength. This result is consistent with the expressible drip of the surimi gel (as depicted in Figure 3).

### 3.4. Umami Amino Acid Evaluation of Reconstituted Surimi Gel

**Figure 4:**
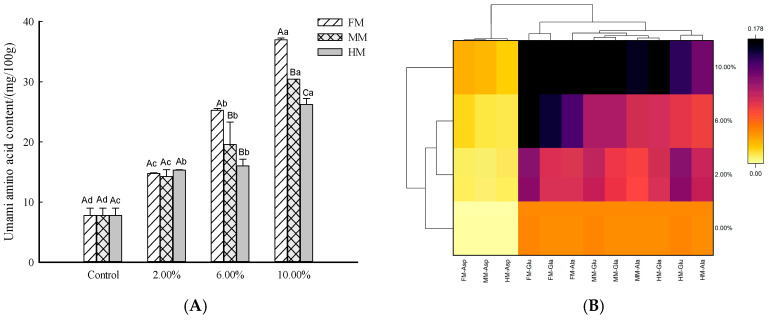
Effects of processing by-products on umami amino acid content in reconstituted surimi gels: bar chart (**A**) and heatmap (**B**). *Note:* a–c indicate significant differences among the additions of the same additive (*p* < 0.05) according to Duncan’s test. A–C indicate significant differences among the additives at the same quantity (*p* < 0.05) according to Duncan’s test.

The release of flavor in surimi products is linked to how flavor substances distribute and diffuse within their three-dimensional network structure. Additionally, the preservation and spread of flavor components are intricately tied to the characteristics of this network structure, and the interactions between the flavor substances and the gel network. As shown in Figure 4B, upon introducing FM, MM, and HM into the mix, the significant intensifications in colorimetric regions FM, MM, and HM indicate markedly increased umami amino acid contents (*p* < 0.05, Figure 4A). In Xu’s study, the results show that the concentrations of amino acid nucleotides and organic acids changed after adding chicken breast. This provides a theoretical basis for the development of the sturgeon processing industry and surimi products from the perspective of lipid changes [28].

Judging from the color changes, the increases in the FM and MM groups were significantly more pronounced (Figure 4B). When the added amounts were 2.00% and 6.00%, there was no significant difference in the HM group, and after reaching 2.00%, the content ranking was FM > MM > HM (Figure 4). According to previous research results, the content of umami amino acids in the large yellow croaker muscle (61.70 mg/100 g) was significantly greater than that in minced meat (46.36 mg/100 g) and fish head meat (45.81 mg/100 g). With an increase in the amount of the water-soluble substances, the expressible drip and cooking loss of the reconstituted surimi gel were increased (as depicted in Figure 3). These factors might have led to a decrease in the umami amino acid content, but the total umami amino acid content of each group still showed an increase. In addition, this may indicate that the water-soluble taste substances extracted from by-products had a stronger impact than external forces (centrifugal force and heating) applied to them.

### 3.5. Low-Field NMR Spin–Spin Relaxation (T_2_)

Low-field nuclear magnetic resonance technology (LF-NMR) is a widely adopted and efficient method for rapidly conducting non-destructive analyses of water distribution and migration within food samples. Its application in the field enables researchers to gain valuable insights into the behavior of water in food matrices. The fitted transverse relaxation time (T_2_) serves as a valuable metric for understanding water mobility and water–protein interactions within the surimi gels. Simultaneously, the area under the curve in the T_2_ spectrum offers valuable insights into the quantity of water present within each gel component [21,22,23,24,25,26,27,28,29].

**Figure 5:**
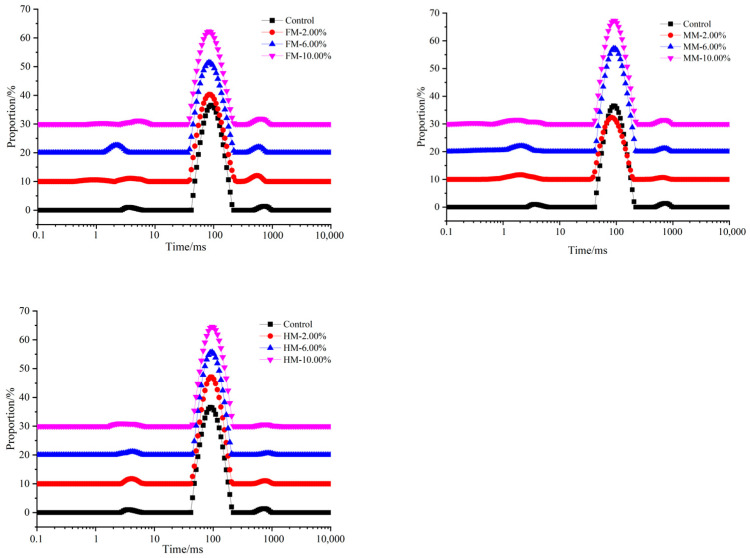
Analysis of relaxation times of reconstituted surimi gels.

After heat treatment, as shown in Figure 5, the relaxation time distributions in different reconstituted surimi gels also indicated three water populations: T_21_ (1~10 ms; bound water) represents water interacting with macromolecules; T_22_ (10~100 ms; immobile water) represents water in the protein gel network, and T_23_ (100~1000 ms; free water) represents water that exists outside the protein lattice. However, three relaxation components were observed in surimi (as depicted in Table 1)—T_2b_ (0~1 ms; strongly bound water), T_21_ (1~10 ms; weakly bound water) and T_22_ (100~150 ms; immobile water). The T_21_ value of the 10.00% FM group (1.24 ms) exhibited a smaller magnitude compared to that of the 10.00% MM and 10.00% HM groups. Notably, in these groups, the T_21_ range appeared relatively wide, indicating a greater extent of interactions between protein aggregates and an increased level of hydration between proteins and water [30]. In general, the T_21_ peak of the surimi gels exhibited a leftward shift, indicating a reduction in relaxation time. This phenomenon can be attributed to the inclusion of WTST, which facilitates the unfolding and cross-linking of protein molecules. As a result, a more condensed and stable network structure is formed, enabling the greater retention and stabilization of water molecules within the gel network [31]. The longer relaxation time indicates the presence of more mobile water in the gel structure, and represents that the water is less tightly bound to the protein. In Singh A’s study, the shortening of the binding water relaxation time after adding sodium bicarbonate (SB) indicates that the binding ability of fish paste protein and water is stronger. The addition of SB improves the ability of the gel to bind water molecules, and thus improves its water holding capacity [32].

As depicted in the Figure 5, in the reconstituted surimi, the contents of combined water (P2b and P21) and immobile water (P22) were detected. The bound water and immobile water accounted for the main part, and the relative content of immobile water (P22) is shown in Figure 5.

### 3.6. Sensory Evaluation Analysis

**Figure 6:**
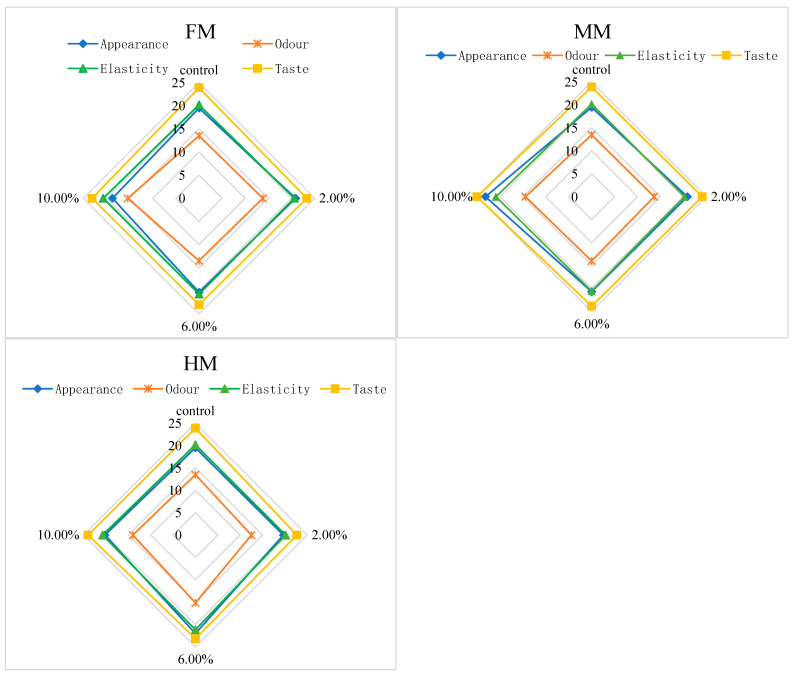
Sensory scores of the reconstituted surimi gel.

**Figure 7:**
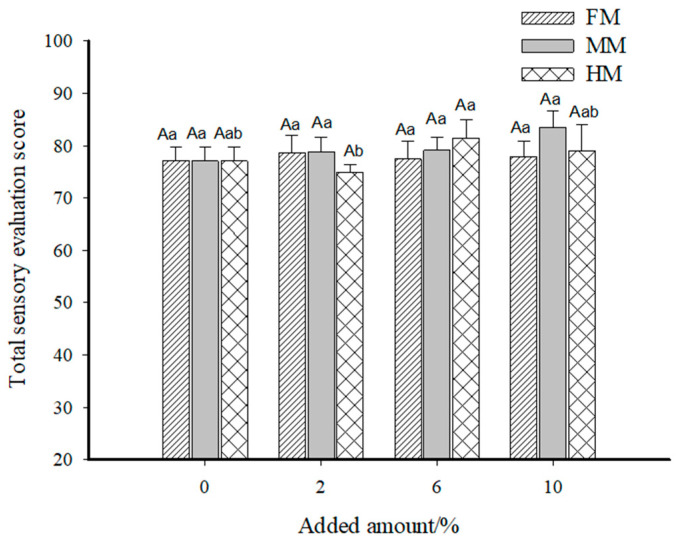
Total sensory scores of the reconstituted surimi gel. *Note:* a–b indicate significant differences among the additions of the same additive (*p* < 0.05) according to Duncan’s test. A indicates significant differences among the additives at the same quantity (*p* < 0.05) according to Duncan’s test.

During the sensory evaluation process, panelists were required to chew and taste the food in their mouth for a certain period before swallowing. This process aimed to promote the thorough mixing of the food with oral saliva, so as to fully release the flavor components in the food, and to simultaneously allow them to perceive the textural properties of the food. Figure 6 shows the sensory scores obtained for each sample under each evaluation indicator, and the analysis was conducted in combination with the total sensory scores shown in Figure 7.

Based on the sensory scores of each index in the FM group, the overall appearance and mouthfeel achieved the highest scores of 20.90 and 23.30, respectively, at an addition level of 2.00%. When the addition level reached 10.00%, the odor score of the surimi gel in the FM group peaked at 15.40, while no significant differences were observed in elasticity among groups. Combining the total sensory scores of the FM group in Figure 7, we see that the surimi gel reached the optimal overall sensory values at 2.00% addition, with a total score of 78.60. This phenomenon can be attributed to the fact that higher addition levels increased the hardness and chewiness of the surimi gel. For the same chewing time, the release of overall flavor and the texture properties of surimi products were relatively reduced, leading to lower total sensory scores. The sensory evaluations of the MM and HM groups were conducted using the 2.00% FM group as the reference standard. For the MM group, the highest scores for appearance, odor, elasticity, and mouthfeel (23.14, 14.43, 20.86, and 25.00, respectively) were obtained at an addition level of 10.00%, resulting in the highest total sensory score of 83.43. In the HM group, the highest scores for appearance, odor, and elasticity (22.00, 15.14, and 21.14, respectively) were observed at 6.00% addition, while the highest mouthfeel score occurred at 10.00%. However, as shown in Figure 7, the highest total sensory score for the HM group was 81.43 at 6.00% addition.

Overall, the sensory performances of surimi gels with water-soluble flavor substances were superior to that of the 2.00% FM group. This is primarily related to the state of exogenous additives in the surimi system—the large yellow croaker muscle filler increased gel hardness and chewiness to some extent, whereas the liquid-state water-soluble flavor substances produced surimi gels with relatively soft textures, facilitating chewing and flavor release.

## 4. Conclusions

This work has shown that *P. crocea* muscle and water-soluble taste substances could replace the originally used by-products as taste enhancers, and could be added to freshwater surimi to prepare reconstituted surimi gel with good gel characteristics and a delicious taste. In the reconstituted surimi gel, the 10.00% FM, 10.00% MM, and 10.00% HM groups exhibited the smallest particle sizes and shorter bound water relaxation times, indicating that protein molecules, lipid molecules, water molecules, and other molecules are tightly bound in these gels. Besides this, the umami taste was well distributed in the three-dimensional network structure, improving the mouthfeel. Therefore, in the context of the comprehensive utilization of raw materials, our current research results provide theoretical guidance for the processing of reconstituted surimi gel, and provide a reference method of relevance to further research into freshwater surimi products with seafood flavors in the future.

## Figures and Tables

**Table 1:** Effects of processing by-products on the particle sizes of reconstituted surimi.

Particle Size/μm	Treatment	FM/μm	MM/μm	HM/μm
D10	Control	7.88 ± 0.04 ^Ac^	7.88 ± 0.04 ^Ac^	7.88 ± 0.04 ^Ac^
	2.00%	8.45 ± 0.24 ^Cb^	13.08 ± 0.24 ^Aa^	9.77 ± 0.11 ^Ba^
	6.00%	9.74 ± 0.12 ^Ba^	13.07 ± 0.14 ^Aa^	8.23 ± 0.05 ^Cb^
	10.00%	6.72 ± 0.22 ^Cd^	12.05 ± 0.25 ^Ab^	7.47 ± 0.11 ^Bd^
D50	Control	20.93 ± 0.17 ^Ad^	20.93 ± 0.17 ^Ad^	20.93 ± 0.17 ^Ad^
	2.00%	70.57 ± 2.80 ^Ab^	57.61 ± 1.07 ^Ba^	52.53 ± 1.45 ^Ca^
	6.00%	105.37 ± 1.57 ^Aa^	51.66 ± 1.79 ^Bb^	49.08 ± 5.29 ^Bb^
	10.00%	38.17 ± 3.77 ^Bc^	47.24 ± 1.32 ^Ac^	38.92 ± 0.66 ^Bc^
D90	Control	93.34 ± 0.59 ^Ad^	93.34 ± 0.59 ^Ac^	93.34 ± 0.59 ^Ac^
	2.00%	242.10± 23.82 ^Ab^	154.97 ± 1.82 ^Cb^	190.90± 2.91 ^Ba^
	6.00%	284.10 ± 6.71 ^Aa^	156.60 ± 5.91 ^Cb^	195.97 ± 4.85 ^Ba^
	10.00%	203.27 ± 15.01 ^Ac^	164.80 ± 2.27 ^Ba^	169.37 ± 1.85 ^Bb^
d4, 3	Control	36.59 ± 0.19 ^Ad^	36.59 ± 0.19 ^Ad^	36.59 ± 0.19 ^Ac^
	2.00%	103.39 ± 10.23 ^Ab^	72.46 ± 1.08 ^Ba^	81.88 ± 2.72 ^Ba^
	6.00%	127.53 ± 2.22 ^Aa^	70.39 ± 2.58 ^Cab^	80.91 ± 1.28 ^Ba^
	10.00%	78.51 ± 6.63 ^Ac^	68.37 ± 1.12 ^Bb^	67.83 ± 0.66 ^Bb^
d3, 2	Control	11.27 ± 0.22 ^Ad^	11.27 ± 0.22 ^Ac^	11.27 ± 0.22 ^Ab^
	2.00%	16.25 ± 0.51 ^Bb^	26.94 ± 0.36 ^Aa^	16.34 ± 1.41 ^Ba^
	6.00%	17.59 ± 0.41 ^Ba^	22.35 ± 0.27 ^Ab^	15.14 ± 0.15 ^Ca^
	10.00%	13.70 ± 0.46 ^Cc^	22.25 ± 0.76 ^Ab^	15.00 ± 0.25 ^Ba^

***Note*:** Mean values of six replicates and standard deviation. FM: large yellow croaker muscle. MM: water-soluble substance concentrated three times from fish skin and fish tail minced meat. HM: the water-soluble substance concentrated two times from fish head meat. a–d indicate significant differences among the additions of the same additive (*p* < 0.05) according to Duncan’s test. A–C indicate significant differences among the additives at the same quantity (*p* < 0.05) according to Duncan’s test. The same is shown below.

**Table 2:** Effect of processing by-product on gel strength and TPA of reconstituted surimi gel.

Treatment		Gel Strength/(g*mm)	Hardness/g	Springiness/mm	Gumminess/mJ	Chewiness/mJ
**FM**	Control	2024.34 ± 121.46 ^Ac^	638.84 ± 17.07 ^Ac^	0.95 ± 0.01 ^Ab^	539.69 ± 13.30 ^Ac^	513.67 ± 10.38 ^Ac^
	2.00%	2451.62 ± 139.24 ^Ab^	716.52 ± 61.97 ^Ab^	0.97 ± 0.01 ^Aa^	605.40 ± 50.17 ^Ab^	590.03 ± 46.21 ^Ab^
	6.00%	3009.12 ± 55.67 ^Aa^	757.73 ± 69.10 ^Ab^	0.97 ± 0.01 ^Ba^	644.10± 55.54 ^Aab^	623.97 ± 52.91 ^Aab^
	10.00%	3244.70 ± 144.30 ^Aa^	831.52 ± 39.21 ^Aa^	0.98 ± 0.01 ^Aa^	683.63 ± 33.22 ^Aa^	666.30 ± 37.32 ^Aa^
**MM**	Control	2024.34 ± 121.46 ^Ac^	638.84 ± 17.07 ^Ac^	0.95 ± 0.01 ^Aa^	539.69 ± 13.30 ^Ad^	513.67 ± 10.38 ^Ad^
	2.00%	2407.22 ± 90.44 ^Ab^	579.95 ± 36.34 ^Bb^	0.92 ± 0.15 ^Aa^	489.60 ± 29.55 ^Bc^	482.64 ± 28.20 ^Bc^
	6.00%	2500.28 ± 55.67 ^Cab^	732.54 ± 16.67 ^ABa^	0.97 ± 0.01 ^ABa^	615.87 ± 14.00 ^ABb^	601.11 ± 19.74 ^Bb^
	10.00%	2670.82 ± 210.42 ^Ba^	761.76 ± 24.55 ^Aa^	0.97 ± 0.01 ^ABa^	656.35 ± 28.75 ^Aa^	635.79 ± 31.03 ^Aa^
**HM**	Control	2024.34 ± 121.46 ^Ad^	638.84 ± 17.07 ^Aab^	0.95 ± 0.01 ^Ac^	539.69 ± 13.30 ^Aab^	513.67 ± 10.38 ^Ab^
	2.00%	2471.81 ± 104.58 ^Ac^	589.47 ± 60.98 ^Bb^	0.97 ± 0.01 ^Aa^	499.67 ± 49.51 ^Bb^	493.11 ± 43.38 ^Bb^
	6.00%	2915.57 ± 107.55 ^Bb^	683.47 ± 9.06 ^Bab^	0.97 ± 0.01 ^Aa^	576.54 ± 13.95 ^Bab^	556.66 ± 25.14 ^Bab^
	10.00%	3214.82 ± 228.48 ^Aa^	759.44 ± 150.10 ^Aa^	0.98 ± 0.01 ^Ab^	650.75 ± 121.21 ^Aa^	628.14 ± 114.78 ^Aa^

***Note*:** a–d indicate significant differences among the additions of the same additive (*p* < 0.05) according to Duncan’s test. A–C indicate significant differences among the additives at the same quantity (*p* < 0.05) according to Duncan’s test.

## Data Availability

The original contributions presented in the study are included in the article, and further inquiries can be directed to the corresponding authors.
